# Supplementary material for: Physiological, Hormonal and Metabolic Responses of two Alfalfa Cultivars with Contrasting Responses to Drought
Source: Int J Mol Sci. 2019 Oct 15;20(20):5099. doi: 10.3390/ijms20205099 (PMC6829892; doi:10.3390/ijms20205099)
Supplement: Supplementary file 1 [file ijms-20-05099-s001.pdf]

Table S1. Preliminary study measures of biomass, gas exchange and mineral composition under well-watered (WW) and water-stressed conditions (WS) in *Medicago sativa* plants. Gas exchange determinations: photosynthetic rates (A), transpiration (E), stomatal conductance (gs), and substomatic CO_2 concentration (Ci). Each value represents the mean ±SE (n=6). The different letters indicate significant differences ($p < 0.05$).

			Gas exchange measures				Mineral composition and isotopes		
		**Total Biomass (g DW plant^{-1})**	**A (µmol m^{-2} s^{-1})**	**E (mmol H_2O m^{-2} s^{-1})**	**gs (mmol H2O m^{-2} s^{-1})**	Ci (µmol mol^{-1})	Leaf N (%)	Leaf C (%)	Carbon isotope fraction (δ ^{18}O)
**San Isidro**	WW	1.69 ± 0.39	10.83 ± 1.38	1.49 ± 0.18	111.2 ± 13.69	237.6 ± 4.32	2.60 ± 0.07	42.38 ± 0.34	-33.23 ± 0.27
	WS	1.64 ± 0.26	2.74 ± 0.52	0.29 ± 0.07	22.04 ± 5.04	180.1 ± 28.73	2.73 ± 0.11	41.05 ± 0.49	-32.78 ± 0.18
**Zhong Mu**	WW	1.96 ± 0.46	8.63 ± 0.67	1.23 ± 0.06	92.13 ± 4.58	245.0 ± 5.54	2.74 ± 0.16	42.47 ± 0.20	-33.48 ± 0.20
	WS	1.23 ± 0.38	4.85 ± 0.59	0.51 ± 0.08	38.53 ± 6.74	181.7 ± 20.68	2.69 ± 0.15	42.18 ± 0.35	-32.65 ± 0.31
Giulia	WW	1.89 ± 0.61	9.81 ± 0.47	1.38 ± 0.05	105.8 ± 4.91	241.9 ± 12.34	3.39 ± 0.15	42.50 ± 0.48	-33.13 ± 0.29
	WS	1.67 ± 0.39	3.75 ± 0.60	0.49 ± 0.08	36.60 ± 5.92	231.0 ± 7.93	2.58 ± 0.12	41.73 ± 0.44	-32.89 ± 0.18
Europa	WW	1.31 ± 0.26	7.84 ± 1.12	1.06 ± 0.17	81.49 ± 13.08	235.3 ± 15.69	3.11 ± 0.10	42.68 ± 0.40	-33.24 ± 0.30
	WS	1.54 ± 0.28	2.74 ± 0.58	0.38 ± 0.08	28.40 ± 6.05	241.3 ± 2.12	2.69 ± 0.20	41.72 ± 0.30	-32.92 ± 0.27
Aragón	WW	2.14 ± 0.31	8.11 ± 0.65	1.32 ± 0.18	101.7 ± 15.65	258.9 ± 11.67	2.82 ± 0.20	41.80 ± 0.47	-33.49 ± 0.18
	WS	1.75 ± 0.30	2.30 ± 0.76	0.28 ± 0.14	21.22 ± 10.69	212.0 ± 23.25	2.98 ± 0.21	41.43 ± 0.29	-32.90 ± 0.17
Long Mu 803	WW	1.51 ± 0.26	8.05 ± 1.09	1.18 ± 0.16	89.40 ± 13.77	247.7 ± 3.63	2.90 ± 0.11	41.83 ± 0.25	-33.41 ± 0.14
	WS	1.84 ± 0.43	2.28 ± 0.80	0.27 ± 0.14	20.75 ± 10.36	225.7 ± 50.79	3.08 ± 0.12	41.32 ± 0.15	-32.85 ± 0.14
Dongmung I	WW	1.62 ± 0.45	9.98 ± 0.93	1.53 ± 0.24	119.1 ± 20.21	253.3 ± 13.35	3.19 ± 0.16	42.24 ± 0.64	-32.77 ± 0.31
	WS	1.74 ± 0.35	2.79 ± 1.04	0.40 ± 0.14	29.93 ± 10.37	276.0 ± 22.99	3.07 ± 0.18	42.10 ± 0.50	-33.22 ± 0.13
Zhao Long	WW	1.56 ± 0.41	10.37 ± 0.45	1.52 ± 0.06	115.6 ± 5.07	248.2 ± 11.88	3.26 ± 0.20	41.88 ± 0.40	-32.76 ± 0.35
	WS	1.23 ± 0.18	2.25 ± 0.50	0.36 ± 0.05	26.45 ± 4.03	262.1 ± 14.40	2.77 ± 0.08	41.98 ± 0.26	-33.37 ± 0.23

Table S2. Gas exchange determinations: photosynthetic rates (A), transpiration (E), stomatal conductance (gs), and substomatic CO_2 concentration (Ci) of *Medicago sativa* plants under well-watered (WW) and water-stressed conditions (WS). Measurements were conducted at the end of the experiment. Each value represents the mean ±SE (n = 6). The different letters indicate significant differences ($p < 0.05$).

	**San Isidro**		Zhong Mu	
Gas exchange	**WW**	**WS**	**WW**	**WS**
A (μmol m^{-2} s^{-1})	11.99 ± 0.60 a	3.51 ± 0.40 b	10.45 ± 0.80 a	4.64 ± 0.55 b
E (mmol H_2O m^{-2} s^{-1})	1.64 ± 0.08 a	0.40 ± 0.04 b	1.41 ± 0.07 a	0.58 ± 0.10 b
g_s (mmol H_2O m^{-2} s^{-1})	122.69 ± 6.16 a	29.87 ± 2.63 b	104.31 ± 5.47 a	43.79 ± 7.86 b
C_i (μmol mol^{-1})	235.90 ± 4.34 a	205.94 ± 25.71 b	233.52 ± 6.35 a	205.30 ± 29.59 b
